# Risk Factors and Options to Improve Engraftment in Unrelated Cord Blood Transplantation

**DOI:** 10.4061/2011/610514

**Published:** 2011-04-05

**Authors:** Anna D. Petropoulou, Vanderson Rocha

**Affiliations:** ^1^Université de Paris 7, Hospital Saint-Louis, 1, Avenue Claude Vellefaux, 75010 Paris, France; ^2^Sirio Libanes e ITACI Hospital (Children's Cancer Hospital), Universtity of Sao Paulo, Sao Paulo, Brazil

## Abstract

Use of umbilical unrelated cord-blood (UCB) cells as an alternative source of hematopoietic cell transplantation has been widely used mainly for patients lacking an HLA-matched donor. UCB present many advantages over bone marrow or mobilized peripheral blood from volunteer donors, such as rapid availability, absence of risk for the donor, and decreased incidence of acute graft-versus-host disease. However, a significant clinical problem is delayed engraftment that is directly correlated with the number of hematopoietic stem cells in a cord-blood unit. The identification of prognostic factors associated with engraftment that can be easily modified (e.g., strategies for donor choice) and the development of new approaches including use of multiple donors, intrabone injection of UCB, ex vivo expansion, and cotransplantation with accessory cells are of crucial importance in order to circumvent the problem of delayed engraftment after UCB transplantation. Those approaches may increase the quality and availability of UCB for transplantation.

## 1. Introduction

UCB transplantation has extended the availability of allogeneic hematopoietic cell transplantation (HCT) to patients who would otherwise not be eligible for this curative approach. In comparison with other sources of allogeneic HCT, UCB offers substantial advantages [1], including (i) significantly faster availability of banked cryopreserved UCB units, with patients receiving UCB transplantation in a median of 25–36 days earlier than those receiving an unrelated bone marrow graft, (ii) extension of the donor pool due to tolerance of 1-2 HLA mismatches out of 6, (iii) lower incidence and severity of acute graft-versus-host disease (GvHD), (iv) lower risk of transmitting infections by latent viruses such as cytomegalovirus (CMV) and Epstein-Barr virus (EBV), (v) lack of risk to the donor, and (vi) higher frequency of rare haplotypes compared to bone marrow registries, since it is easier to target ethnic minorities. 

However, the main problem with using UCB for transplantation is the relatively low number of hematopoietic progenitor cells (HPC) and HSC in UCB compared with bone marrow or mobilised peripheral blood (MPB) grafts which translates into increased risk of graft failure, delayed hematopoietic engraftment [2–6], and delayed immune reconstitution [7, 8]. The cumulative incidence of non-engraftment after UCB transplantation varies from 10 to 20% and the median time to neutrophil recovery varies from 22 to 27 days. Many approaches have been investigated to enhance collection of HSC and HPC in cord blood units. Examples include injecting cord blood cells directly into the bone marrow [9], *in vivo* or *ex vivo* amplification of cord blood cells [10, 11], use of double unit UCB transplantation [12, 13], use of reduced intensity conditioning (RIC) regimen [13–15], and coinfusion with a haploidentical T cell depleted graft [16, 17] or mesenchymal stem cells [18]. 

Many prognostic studies for improving engraftment after UCB transplantation have been performed, analyzing factors related to patients, disease, donor, and transplantation [5, 19–25]. Modifiable factors have been identified, such as HLA, cell dose, and others related to the graft choice or factors related to conditioning regimen [26] or GVHD prophylaxis [27]. 

This paper will focus on risk factors affecting engraftment after UCB transplantation and on procedures aiming to guide clinicians to avoid graft failure following UCB transplantation.

## 2. Risk Factors for Engraftment

### 2.1. Impact of Cell Dose, HLA, and Diagnosis

Almost all series concerning UCB transplantation in children and adults have demonstrated the profound impact of cell dose, measured as prefreezing or infused total nucleated cells (TNC), colony-forming cells, and CD34^+^ cells on engraftment, transplant-related events, and survival [28]. HLA matching was also recognized as an important factor for engraftment. In 1997, Eurocord group has described for the first time the association of TNC dose and HLA with neutrophils and platelets recovery and survival, in 143 patients, mostly children, given a related and unrelated cord-blood transplantation [19]. In fact the median TNC dose infused (3.7 × 10^7^/kg) was the best cutoff value that was associated with higher probability of neutrophils and platelets recovery and improved survival rate. Furthermore, a better HLA matching (defined as matched or mismatched based on HLA-A and -B low-resolution and HLA-DRB1 high-resolution typing) was also associated with better engraftment and survival, but due to small number of patients, the number of HLA disparities associated with outcomes was not studied. Later on, those results have been confirmed in a series of 562 children and adults who received unrelated cord-blood cell grafts [20]: higher cell dose and number of HLA disparities (6/6, 5/6, or 4/6, considering the same above HLA definition) were independent factors associated with better engraftment and decreased transplantation-related mortality. According to the aforementioned studies, it was clear that HLA matching and cell dose were crucial factors for improving outcomes after UCB transplantation, and probably the number of TNC collected or infused should not be inferior to 2.5 × 10^7^/kg or 2.0 × 10^7^/kg (considering a loss of TNC around 20%). Also, the number of HLA disparities should be higher or equal to 4 out of 6 (following the above definition).

### 2.2. Transplantation-Related Factors: Conditioning Regimen and GVHD Prophylaxis

Factors related to the technique of transplantation, such as conditioning regimen and GVHD prophylaxis may also be associated with more rapid engraftment. 

In a recent Eurocord study, the use of Fludarabine in myeloablative conditioning regimens was associated with improved neutrophil and platelet recovery in adult UCB transplantation recipients receiving a lower TNC dose [26]. Use of fludarabine in the preparative regimen has also been associated with improved engraftment independently of cell dose and HLA in UCB transplantation for patients with Fanconi anemia [29]. Conversely, the use of methotrexate (MTX) containing regimens for GVHD prophylaxis has been associated with delayed engraftment and increased risk of graft failure in patients with hemoglobinopathies transplanted with an HLA identical sibling cord-blood unit [27]. However, its use in elsewhere in UCB transplantation has not been evaluated. In Europe and the USA, the most common regimen is calcineurin inhibitor-based GVHD prophylaxis alone or in combination with steroids or mycophenolate mofetil. Nevertheless, Japanese transplant centers have shown interesting results with calcineurin inhibitors in combination with low-dose MTX [30, 31]. Prospective studies are needed to establish the role of MTX in GVHD prophylaxis in the setting of UCB transplantation.

## 3. Approaches to Improve Engraftment and Decrease Early Transplantation Mortality

### 3.1. Selection of Cord Blood Units Based on the Interactions between Cell Dose, HLA Disparities, and Diagnosis

Cell dose and HLA disparities are important and independent factors that interact mutually on engraftment following UCB transplantation. 

Eurocord group has tried to analyze the interaction of cell dose and HLA in 550 UCB transplantation recipients, adults and children, with malignant disorders [23]. We have found that 60 day cumulative incidence (CI) of neutrophils engraftment for all patients was 74%, whereas the incidences for those with no HLA disparity (6/6) versus 1 or more out of 6 HLA disparities were 83% and 53%, respectively. The number of HLA disparities was correlated with neutrophils recovery with a log-linear relationship between HLA disparity and risk of graft failure, suggesting inferior engraftment with increased disparity. Cumulative incidence (CI) of neutrophil and platelet recovery was also associated with the number of TNC before freezing and the use of granulocyte colony-stimulating factor. 

Eapen et al. have also tried to analyze the interaction between cell dose and HLA, comparing outcomes of 503 UCB transplantations and 282 unrelated bone marrow transplants (UBMTs) in children with acute leukemia [6]. A cut-off for collected cell dose was defined as 3.0 × 10^7^/kg for survival in children given a 5/6 HLA disparity graft, but a cell dose cutoff associated with survival of children given a 6/6 or 4/6 grafts were not found. The probability of neutrophils recovery by day 42 and platelets by 6 months were similar after UBMT or matched UCB transplantation (6/6). However, higher cell doses (>3.0 × 10^7^/kg) resulted in a higher probability of engraftment in 5/6 UCB transplantation but had no effect in 4/6 UCB transplantation, probably suggesting that cell dose may not be able to overcome the adverse impact of HLA mismatching in the setting of 4/6 UCBT. 

Recently, Barker et al. [32] analyzed the combined impact of prefreeze TNC dose and HLA match upon CBT outcome in recipients of 0 to 3 HLA-A, -B antigen, and -DRB1 allele-mismatched CB units. A thousand and sixty-one patients were studied, having received a single-unit CBT for the treatment of acute leukaemia or myelodysplasia, after myeloablative conditioning. The study demonstrated that the best outcome for neutrophil and platelet engraftment, acute GVHD, transplant-related mortality (TRM), treatment failure, and overall mortality was associated with the transplantation of 6/6 HLA-matched units, regardless of the TNC dose. The next best survival outcomes were observed in recipients of 5/6 HLA match with a TNC dose of 2.5 × 10^7^/kg or greater or 4/6 HLA match units with a TNC dose of 5.0 × 10^7^/kg or greater [32]. 

In both previous analyses, the interaction between cell dose and HLA were studied in patients with malignant disorders. However, other factors such as diagnosis have an important role in rate of engraftment and other outcomes. This is due to the fact that most patients with hemoglobinopathies have a full marrow and have not received chemotherapy or immunosuppression before conditioning, or, in the cases of aplastic anemia, they have often received previous multiple transfusions or had a severe infection at the time of transplantation, thus increasing the risk of nonengraftment. Recently, in the light of the observation that requirements regarding cell dose and HLA matching may differ in malignant and nonmalignant diseases [1], we attempted to construct an algorithm to guide clinicians in choosing the “best” cord-blood unit, taking into account the impact of diagnosis, cell dose, and HLA incompatibilities, in patients receiving a single UCB transplantation. If the cell dose with a single unit is not achieved, a double cord blood transplant should be investigated. With this objective, two different cohorts of patients who had received a single UCB transplantation between 1994 and 2005 were analyzed: 925 patients had a malignant disease and 279 had a nonmalignant disease (Eurocord; unpublished data). Donor-recipient histocompatibility was determined by serology or antigen typing (low resolution) for HLA-A and HLA-B and by allele typing for HLA-DRB1. In the malignant disease group, we found that cell dose was the most important factor influencing outcome; a minimum cell dose of 3 × 10^7^ TNC/kg at collection and of 2 × 10^7^ TNC/kg at infusion needed to be targeted. We also showed that the number of HLA mismatches increased the risk of delayed engraftment and led to a higher incidence of transplant-related mortality (TRM) and chronic GvHD; however, it decreased the risk of relapse, resulting overall in a lack of influence of HLA mismatching on overall survival (OS) and disease free survival (DFS). Type of HLA mismatch did not influence outcome, but matching for HLA-DRB1 appeared better for patients receiving a graft that had two HLA incompatibilities. As stated earlier, increasing cell dose abrogated the effect of HLA mismatching, but not for grafts with 3 or 4 HLA incompatibilities.

Thus, patients with a nonmalignant disease should receive a higher cell dose to obtain engraftment than patients with a malignant disease; this should not be below 4.9 × 10^7^  TNC/kg at collection and 3.5 × 10^7^ TNC/kg at infusion. In nonmalignant disorders, HLA mismatching played a major role in engraftment, GvHD, TRM, and survival, which was partially abrogated by increasing cell dose. A UCB graft containing two or more HLA disparities with a cell dose inferior to 3.5 × 10^7^ TNC/kg should be avoided. Experience of double cord blood transplantation in nonmalignant disorders is still too limited to allow routine recommendation of this type of transplant [33].

### 3.2. Double UCB Transplantation

Because cell dose is considered to be a critical determinant of outcomes in UCB transplantation, the Minneapolis group has demonstrated that transplantation of two partially HLA-matched cord units may overcome the problem of cell dose and make the transplantation of heavier adult patients feasible. This strategy has led to an increased number of adult patients receiving UCB transplantation. Results with double cord-blood transplantation support the safety of the procedure [12, 13]. Chimerism data from these studies reveal that typically only one cord-blood engrafts. In spite of the fact that double cord-blood transplant recipients are heavier than patients receiving a single unit, cumulative incidence of neutrophil recovery does not differ statistically between the two groups. This observation suggests a “booster” effect from the nonengrafting unit. 

#### 3.2.1. Outcomes after Double UCBT Compared with Single UCBT in Adults with Acute Leukemia

Recent data from the Minnesota group suggests that double UCBT is associated with a higher incidence of acute GVHD compared to that of single UCBT but without an increase in nonrelapse mortality. Interestingly, in an analysis of 177 patients with acute leukemia, relapse was significantly lower for early stage (CR1-2) patients who received two UCB units, suggesting a greater GVL effect. DFS was 40% and 51% for single and double unit recipients, respectively (*P* = .35) [34]. 

The Eurocord group has published results of single and double UCBT for patients with lymphoid malignancies [35]. Relapse incidence was reduced for patients transplanted with a dUCBT compared to those patients transplanted with a single unit. Recently, in a preliminary and unpublished analysis, Eurocord in collaboration with the Acute Leukemia Working party of EBMT have compared the outcomes after dUCBT (*n* = 213) with single UCBT (sUCBT = 378) in adult patients with acute myeloid or lymphoblastic leukemia in remission. There were some differences between the two groups: double UCBT recipients were heavier (median weight: 69 kg versus 64 kg, *P* < .01), tended to be older (median age was 39 years versus 36 years, *P* = .09), transplanted more recently (*P* < .01), and more frequently given RIC (55% versus 32%, *P* < .001) and less ATG/ALG (38% versus 61%, *P* < .001) compared to sUCBT recipients, respectively. As expected, double UCBT recipients received a graft containing a higher nucleated cell dose (median of 3.7 × 10^7^/kg versus 2.6 × 10^7^/kg; *P* < .0001) and greater number of HLA disparities (4/6: 72% versus 62%, *P* = .03) when compared to single UCB grafts. There were no differences in the type of leukemia (AML or ALL) or disease status at transplant (53% were in first CR and 47% in CR2 or more). There were no statistical differences in CI of neutrophil recovery (86 ± 3% in double UCBT versus 87 ± 2% in single UCBT, *P* = .62); however, CI of acute GVHD (II–IV) was higher after double UCBT (36 ± 3% versus 25 ± 2%, *P* = .004) compared with single UCBT, but chronic GVHD was not statistically different between the two groups. Two-year CI of NRM was 37 ± 4% after double UCBT, and it was 36 ± 3% after single UCBT (*P* = .62), whereas relapse incidence was reduced after double UCBT (18 ± 3% versus 26 ± 3% after single UCBT) (*P* = .05). DFS at 2 years after double UCBT was 45 ± 3%, and after single UCBT, it was 38 ± 3% (*P* = .05). In a multivariate analysis adjusted for the differences outlined above, double UCBT was associated with an increased risk of acute GVHD (II–IV) and decreased incidence of relapse. DFS was not statistically different between double UCBT and single UCBT recipients; however, we observed an improved DFS rate in patients transplanted in first CR with double UCBT. 

This Eurocord preliminary analysis confirms the previous findings of more GVHD, equivalent NRM, and reduced relapse in adult recipients of double UCBT, mainly for those transplanted in early disease status, showing a higher GVL effect. No impact has yet been observed on DFS, perhaps due to relatively short followup at this stage.

#### 3.2.2. Comparison of Double Unrelated Cord-Blood Transplantation with HLA-Matched Sibling and HLA-Matched and Mismatched Unrelated Hematopoietic Stem Cell Transplantation

Recently, C. Brunstein, on behalf of the University of Minnesota and the Fred Hutchinson Cancer Research Center, published an analysis of 536 patients with malignant disease (AML, *n* = 211; ALL, *n* = 236; CML, *n* = 70, MDS, *n* = 19) transplanted with an HLA 8/8 allele matched related (MRD, *n* = 204) or unrelated donor (MUD, *n* = 152), 1 allele mismatched unrelated donors (MMUD, *n* = 52) or double UCB (*n* = 128) [36]. All patients received myeloablative conditioning with cyclophosphamide and TBI with fludarabine administered prior to double UCBT and received GVHD immunosuppression with a calcineurin inhibitor and either methotrexate (MRD, MUD, and MMUD) or mycophenolate mofetil (MRD and double UCBT). While patients' weight and sex distribution and proportion with standard risk disease were similar, double UCBT patients were younger (median age 25 years, MRD 40 years, MUD 31 years, MMUD 31 years; *P* < .01). The proportion of AML and ALL was similar among groups although more CML patients received a MUD or MMUD. The median followup of survivors was 3.1 years (range: 0.3–8.1 years). When transplant outcomes were compared, double UCBT was associated with slower hematopoietic recovery compared to other stem cell sources, with median times to neutrophil and platelet recovery being at least 1 week and 4 weeks longer after double UCBT, respectively. Furthermore, despite greater HLA mismatch, the cumulative incidence of grade II–IV acute GVHD and chronic GVHD were lowest after double UCBT. Despite a reduced risk of GVHD after double UCBT, the risk of relapse was remarkably, low while TRM was elevated, resulting in similar progression-free survival (PFS). In the absence of a MRD donor, using either MUD or double UCB yield encouraging PFS and are promising donor options. These results support the use of HLA 0–2 antigen mismatched double UCBT in patients with hematological malignancies as front line therapy in patients lacking a matched sibling donor. 

### 3.3. Reduced Intensity Conditioning Regimen Prior to Single or Double UCB Transplantation from Unrelated Donors in Adults

Most studies have tested UCB transplantation in the setting of myeloablative conditioning. RIC before UCB transplantation has been increasingly used in order to decrease toxicity, shorten the duration of aplasia and extend the availability of cord-blood transplantation to the elderly or patients who are not eligible for myeloablative conditioning. The Minnesota group has evaluated the efficacy of UCB in the setting of a nonmyeloablative regimen consisting of fludarabine, cyclophosphamide and a single fraction of total body irradiation (200 cGy) with cyclosporine and mycophenolate mofetil for posttransplantation immunoprophylaxis. The target cell dose for the UCB graft was 3.0 × 10^7^ TNC/kg, resulting in the selection of a second partially HLA-matched UCB unit in 85% of patients [13]. One hundred ten patients with hematologic diseases were enrolled. Neutrophil recovery was achieved in 92% at a median of 12 days. One cord blood unit predominated engraftment and none of the following factors were predictive of which unit eventually dominated: total nucleated CD34^+^ and CD3^+^ cell doses, HLA matching, nucleated cell viability, ABO typing, gender match, or order of unit infusion. Transplantation-related mortality was 26% at 3 years. Survival and event-free survival (EFS) at 3 years were 45% and 38%, respectively. 

More recently, the Société Française de Greffe de Moelle-Thérapie Cellulaire (SFGM-TC) in collaboration with Eurocord reported results of 155 consecutive UCB transplantations performed using a RIC regimen with a median followup of 18 months (range 2–56) [37]. Conditioning regimen was as previously described and GVHD prophylaxis consisted in cyclosporine and mycophenolate mofetil. Cumulative incidence of neutrophil engraftment at day +60 was 80 ± 3% with a median time to achieve neutrophils >0.5/L of 20 days; autologous recovery was seen in 14% of the patients. In multivariate analysis, factors independently associated with better neutrophil recovery were CD34 cell dose (>1.2 × 10^5^/kg) (HR 1.51, *P* = .04), HLA compatibility (0-1 versus 2-3) (HR 1.5, *P* = .05), and previous autograft (HR 1.8, *P* < .01). Cumulative incidence of nonrelapse mortality (NRM) was 18 ± 3% at 18 months. The estimated probability of OS and DFS at 18 months was 62 ± 5% and 51 ± 4%, respectively. 

In summary, both studies demonstrated the feasibility of RIC-UCB transplantation and reported encouraging results with this approach. In spite of reducing the duration of aplasia, cumulative incidence of engraftment remains between 80% to 90%. Once again HLA disparity and cell dose played an important role and myeloid engraftment was achieved in 94% when patients received a well HLA-matched (6/6 or 5/6) cord blood unit(s) with a higher CD34 cell dose [37].

### 3.4. Use of Accessory Cells to Improve Engraftment

#### 3.4.1. Cotransplantation of a UCB Unit with Highly Purified CD34^+^ Cells from Haploidentical Family Donors

Phase I-II clinical trials using accessory population(s) to enhance engraftment have been published, with interesting results. The Spanish group developed a strategy of UCB transplantation with coinfusion of a limited number of highly purified mobilized HSC (MHSC) from a HLA unrestricted third party donor (TPD). Short posttransplant periods of neutropenia were generally observed in adults with haematological disorders receiving UCB transplantation with relatively low cell content and 0–3 HLA mismatches after myeloablative conditioning. This shortened neutropenic phase was due to an early and initially predominant engraftment of the TPD-MHSC. After a variable period of double complete TPD + UCB chimerism, final full UCB chimerism was achieved (cumulative incidence >90%) within 100 days. Early recovery of the circulating neutrophils resulting from the “bridge transplant” of the TPD-MHSC reduced the incidence of serious neutropenia-related infections, also facilitating the use of drugs with myelosuppressive side effects to combat other infections. The observed incidence of GVHD and relapses was low, with overall and disease-free survival curves comparable to those of HLA identical sibling transplants [17, 38, 39].

#### 3.4.2. Cotransplantation of an UCB Unit with Haploidentical Parenteral Multipotent Mesenchymal Stromal Cells [18, 40]

MacMillan et al. [18] have reported an attempt to speed hematopoietic recovery in a single-institution phase I-II clinical trial in which *ex vivo* culture-expanded multipotent mesenchymal stromal cells (MSCs) from haploidentical parental donors were infused at the time of UCB transplantation. Fifteen pediatric patients with high-risk acute leukemia were enrolled. Eight patients received MSCs on day 0, with three patients having a second dose infused on day 21. No serious adverse events were observed with any MSC infusion. All eight patients achieved neutrophil engraftment at a median of 19 days. Probability of platelet engraftment was 75%, at a median of 53 days. With a median followup of 6.8 years, five patients remain alive and disease free. In another pilot study [40], *ex vivo *culture-expanded bone marrow MSC from parental donors were infused at the time of the transplantation or the in case of refractory acute GvHD. Nine patients received MSC immediately after CB transplantation and TPD highly purified mobilized HSC. Neither immediate adverse effects nor significant differences in CB engraftment or acute GvHD development were observed. Four patients developed grade II acute GvHD and two steroid-refractory. The last two achieved complete remission after therapeutic infusions of MSC. 

The results of both pilot studies show that infusion of *ex vivo* culture-expanded haploidentical MSCs into unrelated UCB transplantation recipients can be performed safely. Further studies may investigate the role of coinfusion of MSC in order to improve engraftment after UCB transplantation.

### 3.5. Other Experimental Approaches

#### 3.5.1. Enhancing Collection Homing and Expansion of UCB Cells

Because of the limiting numbers of HSCs and HPCs in banked cord blood, the means to (1) enhance collection of cord blood cells [41], (2) enhance the homing and engraftment of HSCs/HPCs [42, 43], and/or (3) enhance the ex vivo or in vivo expansion of these cells could greatly enhance the quality and usefulness of cord-blood cells for transplantation. 

It is possible to substantially enhance the numbers of HPC collected by perfusing the placental vessels after draining the blood from the cord [41], but the practicality of this method for banking remains to be evaluated. If perfusion of the placenta after collection of blood from the cord is untaken, it would need to be done in selected collection centers with well-trained personnel.

There have been a number of efforts to enhance the homing and engraftment capability of HSCs and HPCs. One such means is to inhibit the enzymatic activity of CD26/Dipeptidylpeptidase IV (DPPIV) with small peptides [42, 43]. CD26/DPPIV truncates and inactivates the chemokine stromal cell-derived factor-1 (SDF-1/CXCL12) that binds a receptor, CXCR4. The SDF-1/CXCL12-CXCR4 axis is known to be important in the *in vitro* chemotaxis and *in vivo* homing of mouse and human HSCs. Not only is the truncated SDF-1/CXCL12 inactive but also blocks the actions of the full-length active form of SDF-1/CXCL12. This finding suggested that a means to enhance SDF-1/CXCL12 activity by preventing its truncation by CD26/DPPIV might enhance the homing and engraftment of HSCs and HPCs. Using small peptide inhibitors of CD26/DPPIV such as Diprotin A or Val-Pyr, it was possible to enhance the homing and engrafting capability of mouse bone marrow HSCs into lethally irradiated mice, and human cord blood HSCs into sublethally irradiated NOD/SCID mice [43]. Other mechanisms are under investigation with the aim to improve *ex vivo* expansion of cord blood cells. Phase I/II clinical trials have started to evaluate safety and toxicity of infusing Notch-ligand Delta 1 or copper chelator tetraethylenepentamine (TEPA; StemEx) to induce *ex vivo* expansion of cord blood progenitors in patients with hematologic malignancies [10, 11]. Interestingly, Notch-ligand Delta 1 has also been shown to have an effect on early T-cell expansion and differentiation [44]. Recently, Delaney et al. have described a Notch-mediated *ex vivo* expansion system for human CD34^+^ cord blood progenitors that results in a marked increase in the absolute number of stem/progenitor cells, including those capable of enhance repopulation in the marrow NOD-SCID mice [45]. Moreover, a phase I trial is ongoing, involving transplantation of a nonmanipulated unit along with cord blood progenitors expanded *ex vivo* in the presence of Notch ligand. Ten patients have been enrolled, who underwent double UCBT after myeloablative conditioning for high-risk acute leukemia. Significantly, more rapid myeloid engraftment was observed, with median time to neutrophil recovery (neutrophils 500/*μ*L) being 16 days, faster than would be expected using 2 nonmanipulated units (median time 23 to 26 days or longer in the published literature) [45]. 

Future efforts to expand HSC/HPC *ex vivo* and *in vivo* and to enhance the homing and engrafting capabilities of cord blood cells will likely make use of more in depth information on intracellular signaling molecules and their networks involved in HSC and HPC functions, including self-renewal, proliferative, survival, differentiation, and migration. Further information on the bone marrow microenvironment and how HSC/HPC interact with this microenvironment will permit the development of more effective ways to achieve engraftment.

#### 3.5.2. Enhancing Homing Capacity with Direct Intrabone Marrow Injection of Cord-Blood Cells

The concept of enhancing homing capacity of cord-blood cells through their direct injection into the bone-marrow environment has led some investigators to apply this approach clinically. In mice, it has been suggested that intrabone infusion of CD34^+^ cord-blood cells confers an engraftment advantage 15 times greater than after intravenous infusion, because cell loss during circulation before homing is circumvented [46]. Recently, a phase I/II study [9] was performed to establish the safety and efficacy of the intrabone administration of cord blood cells, measured by the donor-derived neutrophil and platelet engraftment. Thirty two patients presented leukaemia, 14 with advanced disease. HLA-matching was 5/6, 4/6, and 3/6 for 9, 22, and one patient, respectively. Most of the patients received a myeloablative conditioning regimen associated with ATG and only 2 patients received a RIC prior to UCB transplantation. Cord-blood cells were concentrated in four 5-mL syringes and were infused in the superior-posterior iliac crest under rapid general anaesthesia. Median transplanted cell dose was 2.6 × 10^7^/kg. No complications occurred during or after the intrabone infusion of cells. Median time to recovery of neutrophils was 23 days (range 14–44) and median time to recovery of platelets was 36 days (range 16–64). All patients were fully chimeric from 30 days after transplantation to the last followup visit, suggesting early complete donor engraftment. No patient developed grade III-IV acute GVHD. More recently, in a preliminary matched pair analysis comparing patients transplanted with cord blood injected intravenously (IVCB) versus cord blood injected directed into the bone marrow (IBCB) of the iliac crest, IBCB patients (*n* = 50) were matched with 88 IVCB recipients. Cumulative incidence (CI) of neutrophil recovery was 70 ± 5% in IVCB recipients versus 80 ± 6% in the IBCB group (*P* = .27). However, patients receiving IBCB had a higher CI of platelet recovery at day 60 (82 ± 5%) compared to the IVCB group (40 ± 5%; *P* < .0001). Strikingly, the incidence of acute GVHD grade II–IV was 12% in the IBCB group compared to 38% in the IVCB group (*P* = .0001), and the incidence of grade III-IV GVHD was 2% compared to 18%, (*P* < .001) respectively. Overall survival at one year was 67 ± 7% compared to 43 ± 5% (*P* = .07), respectively. In summary, injection of cord-blood cells into the bone marrow appears to significantly reduce the problem of delayed platelet recovery observed after IVCB. The reduced incidence and severity of acute GVHD observed in IBCB patients is intriguing and promising [9].

## 4. Conclusions

Engraftment after UCB transplantation is improving in the recent years, mainly due to better donor choice (cell dose and HLA matching), improvement in supportive care, greater centre experience. Other approaches that improve engraftment after UCB transplantation are being currently developed with very encouraging results. Those approaches may greatly increase the clinical use of cord-blood cells for transplantation.
